# Natural Rubber Films Reinforced with Cellulose and Chitosan Prepared by Latex Aqueous Microdispersion

**DOI:** 10.3390/polym16182652

**Published:** 2024-09-20

**Authors:** Naipaporn Sutipanwihan, Veerapat Kitsawat, Praewpakun Sintharm, Muenduen Phisalaphong

**Affiliations:** Bio-Circular-Green economy Technology & Engineering Center, BCGeTEC, Department of Chemical Engineering, Faculty of Engineering, Chulalongkorn University, Bangkok 10330, Thailand; smile_8e88@hotmail.com (N.S.); kit.veerapat@gmail.com (V.K.); praewpakun.s@gmail.com (P.S.)

**Keywords:** natural rubber, cellulose, chitosan, composite film, microdispersion, latex

## Abstract

In this paper, green composite films comprising natural rubber (NR), cellulose (CE), and chitosan (CS) were successfully fabricated through a simple, facile, cost-effective method in order to improve mechanical, chemical, and antimicrobial properties of NR composite films. Chitosan with a low molecular weight of 30,000–50,000 g/mol (CS-L) and a medium molecular weight of 300,000–500,000 g/mol (CS-M) was used for the fabrication. The composite films were prepared via a latex aqueous microdispersion method with different weight ratios of NR:CE:CS-L/CS-M. Fourier transform infrared spectroscopy (FTIR) results demonstrated strong interactions of hydrogen bonds between CE and CS-L/CS-M in the composite films. The tensile strength and the modulus of the composite films in dried form were found to significantly increase with the reinforcement of CE and CS-L/CS-M. The maximum tensile strength (13.8 MPa) and Young’s modulus (12.7 MPa) were obtained from the composite films reinforced with CE at 10 wt.% and CS-L at 10 wt.%. The high elongation of 500–526% was obtained from the composite films reinforced with CE at 10 wt.% and CS (CS-L or CS-M) at 5.0 wt.%. The modification could also significantly promote antimicrobial activities and chemical resistance against non-polar solvents in the composite films. The NR composite films have potential uses as flexible films for sustainable green packaging.

## 1. Introduction

Plastics are one of the products derived from petroleum hydrocarbons that have been used in many applications worldwide [1,2]. However, plastic waste can create environmental pollution. Accordingly, natural materials have been proposed as substitutes for petroleum-based materials because of their notable advantages, such as renewable resources and biodegradability. In addition, some bioplastics have been reported to have better elasticity and elongation properties than petrochemical plastics [3].

Southern Thailand is the world’s largest producer and exporter of natural rubber (NR) from rubber trees (*Hevea brasiliensis*). NR is a well-known biopolymer for its high elasticity, which is the ability to regain its original shape after being deformed; however, it is easily decomposed by high temperatures, ultraviolet light, or ozone [4,5]. The mechanical properties of pure NR, such as tensile strength and Young’s modulus, are also poorer than those of many synthetic analogues. In addition, it also has low resistance to petroleum and solvents [6]. Therefore, improving physical properties such as hardness, Young’s modulus, and abrasion resistance, as well as other properties like antimicrobial activity, thermal stability, and chemical resistance, is necessary to enhance the performance of NR for extended applications [7]. The mechanical properties of NR composites can be improved by the addition of reinforcing materials. For considerable environmental benefits, the reinforcing materials in NR matrix should be environmentally friendly, biodegradable, and non-toxic. 

In this study, the authors propose to improve mechanical, chemical, and antibacterial properties of NR by reinforcing it with cellulose and chitosan. Cellulose is the most abundant biopolymer on Earth and serves as the primary structural component of the cell walls in green plants. This fibrous substance provides form and rigidity to cell walls. It is a natural polysaccharide polymer consisting of a few hundred to many thousands of β(1–4) linked D-glucose units. Cellulose fibrils are bundles of these linear chains and contain several hydroxyl groups along their structure; therefore, they can form hydrogen bonds with oxygen atoms on the same or neighboring chains, contributing to high strength [8,9]. Previously, research studies of NR reinforcement by using cellulose as biomaterial filler have been reported [9,10,11,12]. The results demonstrated that the increase in tensile strength and Young’s modulus could be attained in agreement with the increase in the amount of cellulose, but the considerable decrease in elongation at break was detected [13]. On the other hand, chitosan is a natural polysaccharide derived from chitin through a deacetylation pathway. It has a very similar chemical structure to cellulose, except that one of the hydroxyl (–OH) groups is substituted by an amine (–NH_2_) group. The synergistic use of both materials is expected to be beneficial because the various functional groups of cellulose and chitosan can form hydrogen bonds with each other [14,15]. The advantageous properties of cellulose and chitosan include renewability, sustainability, non-toxicity, biocompatibility, and biodegradability. The specific functionality of cellulose and chitosan would lead to the generation of new multifunctional composites for a wide range of applications [16,17,18,19,20,21,22]. In addition, from the research on cellulose-chitosan composite films, the addition of chitosan could promote antimicrobial properties [23]. Cellulose-chitosan blended materials exhibit useful properties, such as antibacterial activity, good mechanical characteristics, high adsorption capacities, high porosity, metal ion adsorption, and barrier properties [24]. 

Despite the individual advantages of cellulose and chitosan, the combined impact of these components in NR composites has not been investigated. In this work, to improve mechanical and chemical properties as well as antimicrobial characteristics, NR composites were reinforced with microcellulose fibers using a chitosan solution as a dispersing agent via a latex aqueous microdispersion method. This novel approach is expected to synergistically enhance the mechanical, thermal, antimicrobial, and chemical resistance properties of the composites. The effects of varying cellulose and chitosan loadings, as well as different molecular weights of chitosan, were investigated. The properties of the developed composites were characterized for further consideration of their potential use.

## 2. Materials and Methods

### 2.1. Materials

NR latex (NRL) with 60% dry rubber content (DRC) was purchased from the Rubber Research Institute of Thailand (RRIT, Bangkok, Thailand). Sheets of cellulose from eucalyptus pulp were kindly provided by Teppattana Paper Mill Co., Ltd. (Bangkok, Thailand). The sheets were dried in an oven at 80 °C for 24 h to remove moisture. The microfibrillated cellulose was prepared by crushing the dried cellulose sheet, grinding it using ball mill (PM 100, Haan, Germany), and sieving, respectively. According to our previous analysis, the cellulose particles mainly demonstrated smooth small cylindrical shapes, with lengths and widths around 20–120 μm and 10–50 μm, respectively [25]. Acetic acid solution (AR grade) and ammonium hydroxide solution (25% AR grade) were purchased from Sigma-Aldrich (Thailand) Co., Ltd. (Bangkok, Thailand). Chitosan powders (CS) with a low molecular weight of 30,000–50,000 g/mol (CS-L) and a degree of deacetylation of 90%, and with a medium molecular weight of 300,000–500,000 g/mol (CS-M) and a degree of deacetylation of 90% were used for the fabrication of composite films. The molecular weight (MW) of CS was indicated by the company from which we purchased the chitosan products (TS Agritech company, Pathum Thani, Thailand). The technical sheets for the chitosan (CS-L and CS-M) are provided in the Supplementary Materials. The toluene (AR grade) used in the toluene uptake (TU) test was purchased from Merck (Darmstadt, Germany).

### 2.2. Methods

#### 2.2.1. Preparation of CE Suspension and CS Solution

The cellulose suspension (CE) and chitosan (CS) solution were prepared at concentrations of 5 wt.% and 10 wt.% (weight of CE or CS per weight of dry NR). The suspension of CE was prepared by mixing CE powders in deionized (DI) water and dispersing the particles in the suspension by using ultrasonication at 500 W for 15 min. Then the CE suspension was added with the ammonium hydroxide solution at 2.5% (*v*/*v*). CS was used as a dispersing agent. The CS solution was prepared by dissolving CS powders in an aqueous solution of 2% (*v*/*v*) of acetic acid. CS with high MW had low solubility in dilute acid solution, and the viscosity of the CS solution with high MW was very high. Therefore, it was not suitable to use CS with high MW for the preparation of a homogeneous mixture of CE and NR. Accordingly, in this study, CS with a low MW of 30,000–50,000 g/mol (CS-L) and CS with a medium MW of 300,000–500,000 g/mol (CS-M) were used for the fabrication of composite films. 

#### 2.2.2. Preparation of NR Composite Films

To fabricate NR composite films, 6 g of NRL was slowly added to 20 mL of the CE suspension and 20 mL of the CS solution. Then the mixture was stirred using high-frequency mechanical stirring at room temperature until a homogeneous suspension was obtained. After that, the mixture was poured into a plastic tray and dried in an oven at 40 °C for 24 h.

The CExCS-Ly or CExCS-My films were defined as a modified NR composite film prepared by adding CE at x% *w*/*w* and CS-L or CS-M at y% *w*/*w*. For example, CE5CS-L10 is the NR composite film prepared by adding CE at 5% of the NR dry weight (% *w*/*w*) and CS-L at 10% of the NR dry weight (% *w*/*w*). The compounding formulations of all composites are summarized in Table 1. The particular polymer concentrations were chosen based on the suitable concentrations that provided good dispersion of CE and CS in NRL during the preparation of NR composites.

### 2.3. Characterization

#### 2.3.1. Scanning Electron Microscope (SEM)

Surface and cross-sectional morphologies were observed using a scanning electron microscope and an energy dispersive X-ray spectrometer (SEM-EDS; JEOL JSM-IT-500HR and JEOL, Tokyo, Japan). The dried NR films reinforced with CE and CS were sputtered with a thin layer of gold in a sputter coater (Balzers-SCD 040, Balzers, Liechtenstein) and examined at a magnification of 500× for surface views and 350–400× for cross-section views with an accelerating voltage of 15 kV. 

#### 2.3.2. Fourier Transform Infrared Spectroscopy (FTIR)

The functional groups and possible interaction between CE/CS/NR were determined by Fourier transform infrared (FTIR) spectroscopy (Perkin Elmer, Waltham, MA, USA) in the ranges of 4000–400 cm^−1^ with a resolution of 4 cm^−1^ and a total scan of 64. 

#### 2.3.3. Mechanical Properties Testing

The tensile strength, Young’s modulus, and elongation at break of NR composite films were measured using an Instron 5567 Universal Testing Machine (INSTRON, Norwood, MA, USA) following the testing conditions reported in an ASTM D882 equipped with a 10 kN load. The average values were determined from at least five specimens and reported as average values with standard deviations (SDs). 

Tensile stress is defined as the ratio of the applied force (*F*) on the material to its cross-sectional area (*A*), and tensile strength is the maximum tensile stress that a material can withstand before breaking.

$$Tensile\;stress\;\left(\sigma \right)=\frac{F}{A}$$

Young’s modulus (*Y*) is the elastic modulus when deformation is caused by either tensile or compressive stress. *Y* is defined as the ratio of tensile stress (*σ*) to tensile strain (*ε*). Tensile strain (*ε)* is the extension per unit original length *(dl/l*), where *l* is the original length of the sample and *dl* is the extension length.

$$Y=\frac{\sigma }{\varepsilon }=\frac{F/A}{dl/l}$$

Elongation at break (or fracture strain) is the ratio between the increase in length and the initial length after breakage of the test specimen. The formula is as follows:

$$\%\;Elongation\;at\;break=\frac{\left(Final\;Length-Initial\;Length\right)}{Initial\;Length}\times 100\%$$

#### 2.3.4. Thermogravimetric Analysis (TGA)

The thermal weight changes of CE, CS, NR, and composite films were determined using TGA (Q50 V6.7 Build 203, Universal V4.5A TA Instruments, New Castle, DE, USA) in a nitrogen atmosphere. The scanning range was 30 °C to 600 °C with a heating rate of 10 °C/min. The initial weight of each sample was around 10 mg, and percentage weight loss versus decomposition temperature by TGA analysis was determined.

#### 2.3.5. X-ray Diffraction (XRD) Analysis

The structural information and crystallinity of NR composite films were characterized by X-ray diffraction (XRD, model D8 Discover, Bruker AXS, Karlsruhe, Germany). 

The samples were cut into square-shaped specimens of 4 cm in width and length. The operating voltage and current were 40 kV and 40 mA, respectively. Samples were scanned from 5–40° 2θ using CuKa radiation.

#### 2.3.6. Contact Angle Analysis

Dried rectangular specimens of 40 mm × 40 mm were used for contact angle measurements. The dynamic advancing and receding water contact angles were measured using a contact angle goniometer (Rame-hart, Instrument Co., Succasunna, NJ, USA, model 100-00) under air at room temperature, equipped with a Gilmont syringe and a 24-gauge flat-tipped needle.

#### 2.3.7. Water Absorption Capacity (WAC) Analysis

Water absorption capacity (WAC) was determined by immersing the pre-weight of dry films of 20 mm × 20 mm in DI water at room temperature (30 °C) until equilibrium. Then, the films were removed from the water and blotted out with Kimwipes. The weights of the hydrate films were measured, and the procedure was repeated until there was no further weight change. The WAC of the sample was calculatedusing the following formula:

(1) $$\mathrm{WAC}\;(\%)=\frac{W_{h}-W_{d}}{W_{d}}\times 100\%$$

where W_h_ and W_d_ denote the weights of hydrate and dry films, respectively.

#### 2.3.8. Toluene Uptake (TU) Analysis

Dried specimens of composite films (25 mm × 25 mm) were weighed and immersed in toluene at room temperature. After that, the specimens were weighed. The procedure was repeated until there was no further weight change. The toluene uptake (TU) was calculated by the following formula:

(2) $$\mathrm{TU}\;(\%)=\frac{W_{t}-W_{0}}{W_{0}}\times 100\%$$

where W_0_ and W_t_ denote the weights of films before and after immersion in toluene, respectively.

#### 2.3.9. Antimicrobial Ability Analysis

The antimicrobial ability of CE/CS/NR composite films against *Escherichia coli* (ATCC 8739), *Staphylococcus aureus* (ATCC 6538P), and *Aspergillus niger* (ATCC 1015) were assessed based on the modified JIS Z 2801 method [26,27]. The JIS Z 2801 method tests the ability of antimicrobial surfaces to inhibit the growth of microorganisms or kill them. The films were cut into 5 cm × 5 cm squares and were sterilized with UV light for 2 h, and then 0.4 mL of microbial cell suspension with an initial cell concentration of 2–10 × 10^6^ CFU/mL was applied to each sample. The incubations of *E. coli*, *S. aureus,* and *A. niger* were at 37 °C under aerobic conditions for 24 h. After that, the film samples were subjected to thorough shaking in 10 mL of pH 7.4 phosphate buffer saline (PBS) for elution of microbial suspensions. Subsequently, the PBS solution containing the cells was cultured on agar plates, and incubations were carried out for 5 days at 25 °C for *A. niger* and 24 h at 37 °C for *E. coli* and *S. aureus* before counting the cell colonies. All microbial counts were performed in triplicate. The percentage reduction of microorganisms was calculated using the following formula:

(3) $$\%\;\mathrm{Reduction}\;\mathrm{of}\;\mathrm{microorganisms}=\frac{B-A}{B}\;\times \;100\%$$

A = CFU per milliliter of viable microorganisms after treatment (24 h)

B = CFU per milliliter of viable microorganisms before treatment (0 h)

#### 2.3.10. Statistical Analysis

Statistical analysis was presented as mean ± the standard deviation (SD) and obtained from at least three independent tests. All data were analyzed using Minitab 17 software. Error bars represent SD. The differences were considered to be statistically significant at the value of *p* < 0.05.

## 3. Results and Discussion

### 3.1. Scanning Electron Microscopy (SEM)

**Figure 1:**
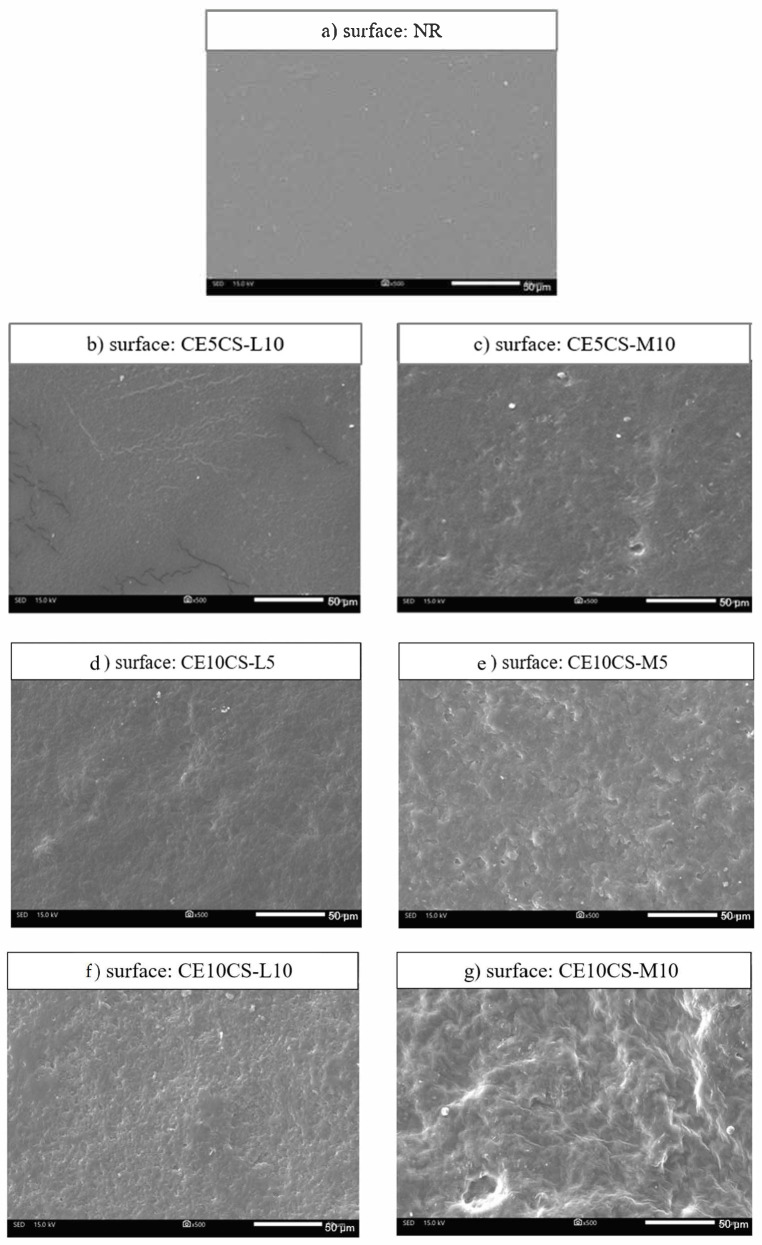
SEM images of the surface views of NR (**a**) and CE/CS/NR composites (**b**–**g**).

Figure 1 shows SEM images of the surfaces of NR and CE/CS/NR films prepared via the latex aqueous microdispersion method [13,28,29]. The thickness of the NR and NR composite films was around 0.3 mm. According to surface SEM images, the neat NR film had a smooth outer surface with a dense structure (Figure 1a), while an increase in roughness was observed on the surfaces of CE/CS/NR films, related to the loading content of CE and CS (Figure 1b–g). The filler, CE, thoroughly dispersed and integrated in the NR matrix and acted as a reinforced filler. The degree of surface roughness increased with increasing contents of CE and CS-M/CS-L. The surface of the films containing CS with medium MW (CE/CS-M/NR) were relatively rougher than those containing CS with low MW (CE/CS-L/NR). The surface roughness could be caused by the differences in polarity and density of NR, CS, and CE [24]. The films of CE/CS-M/NR had rougher surfaces than those of CE/CS-L/NR, as the longer chains of chitosan could increase interactions among neighboring chains. 

**Figure 2:**
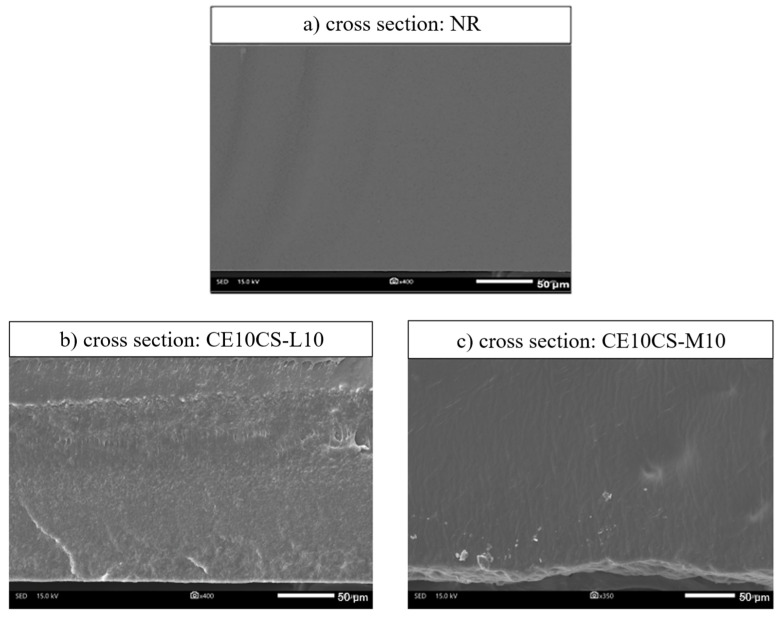
SEM images of the cross-section views of NR (**a**), CE10CS-L10 (**b**) and CE10CS-M10 (**c**).

Figure 2b,c presents the cross-sectional SEM images of the CE/CS/NR films with the highest filler loading (CE at 10 wt.% and CS at 10 wt.%) as compared to the neat NR films (Figure 2a) in order to evaluate the miscibility of fillers in the NR matrix at high concentration loading. In this study, CE and CS suspensions were added and consistently mixed with NR at different weight ratios of 5 and 10 wt.%. By adding cellulose powders (CE) into a chitosan (CS) aqueous solution prior to blending with NRL, a uniform mixture of CE and NRL was observed in a single-phase mixture without any phase-separated compartments. It is suggested that CS in aqueous solution assisted the dispersion of CE in NRL by coating the CE surface and preventing the agglomeration of CE in the NR matrix, leading to the uniform dispersion of CE in NR composite films. Due to the consistent mixing of CE suspension and CS solution in NRL and the strong interfacial interactions from hydrogen bonding between CS chains and CE, a good dispersion of CE and CS in the composites could be obtained [30,31]. The cross-sectional view of the NR film (Figure 2a) shows the homogeneous phase from a single phase without filler dispersion of the pure NR matrix. The cross-sectional views of CE10CS-L10 (Figure 2b) and CE10CS-M10 (Figure 2c) show the composite films with a dense structure. An appropriate incorporation of CE and CS into the NR matrix was observed in the cross-section images of the composite films; only small portions of agglomerated particles appeared even at this concentration. There was no significant difference in the morphology of the samples with the use of CS-L compared to CS-M.

The polarity difference between NR and CE results in poor immiscibility between these two materials, which causes a problem of phase separation or high aggregation of CE when a high amount of CE is used as a reinforcing agent in NR composites [28,29]. In this study, the maximum loadings of CE and CS were 10% and 10%. It was found that under CE loading at low and high concentrations into NR using CS as a dispersing agent, no phase-separated structure was formed, and there was no extensive aggregation of filler particles in the NR matrix of the composite films. Only small white dots were observed on the surface and cross-sectional views of the composite films (Figure 1b–g and Figure 2b,c). However, SEM images taken at higher magnifications are suggested for a more precise evaluation. Overall, the results indicated that the composite films of NR reinforced with CE and CS could be successfully developed using the latex aqueous microdispersion process.

### 3.2. Fourier-Transform Infrared Spectroscopy (FTIR)

**Figure 3:**
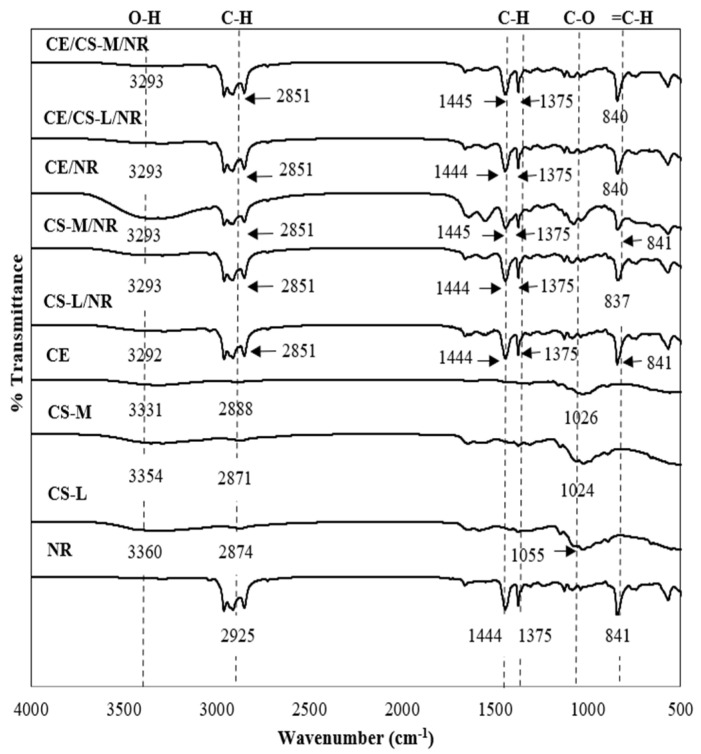
FTIR spectra of NR, CS-L, CS-M, CE, and NR composite films.

Figure 3 demonstrates the FTIR spectra of NR, CS-L, CS-M, CE, and the composite films of CE10/NR, CS-L10/NR, CS-L10/NR, CE10/CS-L10/NR, and CE10/CS-M10/NR. The FTIR spectrum of pure NR shows characteristic peaks at ~2912 cm^−1^, which are attributed to asymmetric stretching vibrations of methylene (–CH_2_), and a peak at 2960 cm^−1^, which is attributed to symmetric stretching vibrations of methyl (–CH_3_) The =C–H out-of-plane bending was observed at 841 cm^−1^ [29,32]. For pure cellulose, there was a strong broad peak at 3331 cm^−1^, which is attributed to O–H stretching vibrations from hydroxyl groups. The peak at 2888 cm^−1^ is attributed to the vibration of C–H stretching [33]. Moreover, there is a peak at 1026 cm^−1^ attributed to C–O symmetric stretching of primary alcohol. In the case of chitosan, the broad and small peak from 3000 to 3600 cm^−1^ is assigned to N–H symmetric stretching of aliphatic primary amine and the stretching vibration of –OH bonds, as well as the stretching C–H group at around 2871 to 2874 cm^−1^ [34,35]. The peak at 1024 cm^−1^ is attributed to C–O symmetric stretching of primary alcohol. The FTIR spectra of CE/CS-L/NR and CE/CS-M/NR composite films show peaks around 3000–3500, 2851–2960, and 841 cm^−1^, which are assigned to N–H, C–H stretching, and C–H out-of-plane bending, respectively. The FTIR results of CS-L/NR and CS-M/NR composite films confirmed the presence of NR and CS. The FTIR results of CE/CS-L/NR and CE/CS-M/NR composite films also show NR, CE and CS peaks, which are around 2851–2960, 1445, 1437, and 841 cm^−1^, assigned to C–H stretching of alkane, C–H stretching vibration of methyl (–CH_3_), O–H stretching of alcohol, and C–H out-of-plane bending, respectively. The broad band peak at 3000–3500 cm^−1^ overlapped by the stretching vibration of the hydroxyl groups and primary amine and all the shifts of the absorption bands indicated that hydrogen bonding, which is a strong interfacial interaction between fillers and polymer matrixes, formed between the hydroxyl (OH) and amine (NH_2_) groups of cellulose and chitosan [30]. The position peaks of all composite films display characteristic peaks of CE, CS, and NR.

### 3.3. Mechanical Properties

**Figure 4:**
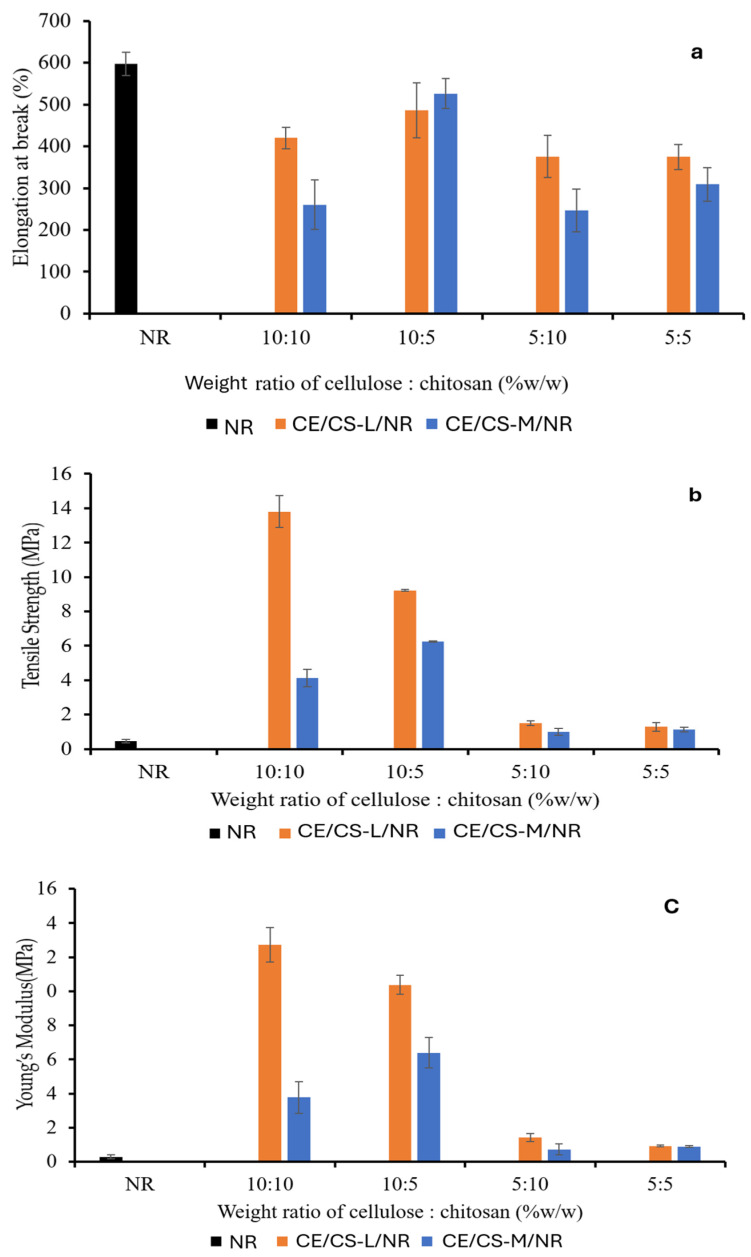
Elongation at break (**a**), tensile strength (**b**), and Young’s modulus (**c**) of composite films as a function of the weight ratios of cellulose (CE) and chitosan (CS-L and CS-M) in the composites.

The mechanical properties of CE/CS-L/NR and CE/CS-M/NR composite films at the different ratios of CE and CS were analyzed in terms of elongation at break, tensile strength, and Young’s modulus, as shown in Figure 4. In general, NR is well-known for its high elasticity [36], whereas CE and CS have high tensile strength and Young’s modulus [37]. The tensile strength and Young’s modulus of the NR composite films were therefore enhanced with reinforcement using CE and CS in suitable proportions with good dispersion of CE and CS in an NR matrix, whereas the elongation values of the composite films decreased compared to the neat NR film. In this work, the maximum tensile strength (13.8 MPa) and Young’s modulus (12.74 MPa) were obtained from the composite films reinforced with CE at 10 wt.% and CS-L at 10 wt.%. The elongation at 500–526% was obtained from the composite films reinforced with CE at 10 wt.% and CS at 5 wt.%, which was relatively lower than that of the neat NR film. The improved mechanical strength is likely due to the inter- and intra-molecular interactions of hydroxyl and amine groups through hydrogen bonding [23,24]. From the study, it was shown that the addition of cellulose (CE) had a greater influence on mechanical properties than the addition of chitosan (CS). However, by using CS as the filler dispersing agent, CE at 5–10%wt could disperse homogeneously within the NR matrix, resulting in significantly improved tensile strength and Young’s modulus of the composite films due to the strong interfacial interactions from hydrogen bonding between CS chains and CE [30].

The CE/CS/NR composite films were less extendable compared with pure NR due to the reduced elongation. In Figure 4a, with increasing chitosan content, the elongation values decreased. When the percentage of chitosan increases, the chain flexibility of system was more limited [38], and the molecular mobility of NR was reduced [39], resulting in the decrease in elongation values. CE has high tensile strength due to firm hydrogen bonds between the individual chains in CE microfibrils. From previous studies, with the integration of CE in the NR matrix, significant improvements in the Young’s modulus and tensile strength of the composites were achieved [40]. 

### 3.4. Thermogravimetric Analysis (TGA) and Derivative Thermogravimetry (DTG)

**Figure 5:**
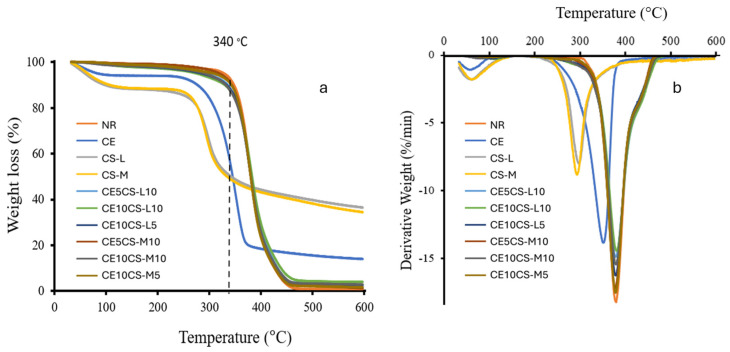
The curves of the thermogravimetric analysis (TGA) (**a**) and the derivative thermogravimetry (DTG) (**b**) of NR, CE, CS, and CE/CS/NR composite films.

The thermal degradation analysis (TGA) was determined, as shown in Figure 5a. The TGA curves show the % weight loss of CE, NR, CS, and CE/CS/NR composite films at various temperatures. The weight loss detected below 200 °C was from the vaporization of water from CE and CS, resulting in a weight loss of approximately 5–12% of the initial weight. The CS, CE, and NR decomposed first, with an initial decomposition of 250, 260, and 340 °C, respectively [25]. The composite films of CE/CS/NR showed trend lines of the polymer decomposition, similar to that of NR. The maximum rate of weight loss in the composite films occurs at around 250–450 °C in accordance with the decomposition of CS, CE, and NR, respectively. At temperatures above 450 °C, the remaining dry weights of the decomposition products, such as char, non-volatile residues with high carbon content, were determined. The char yields of CE10CS-L10, CE10CS-M10, CE10CS-L5, CE10CS-M5, CE5CS-L10, and CE5CS-M10 were 7.65%, 4.81, 5.13%, 1.57%, 3.77%, and 1.10%, respectively. The char yield was higher when the CE and CS content increased. CE and CS are composed of glucose units joined via β-1,4 glycosidic linkage, in which glucose consists of six carbon atoms bonded together as a chain with additional atoms of oxygen and hydrogen. In this study, the crystallinity value of CS-L was 76%, which was higher than that of CS-M (63%). It was demonstrated that the char yields of CS-L and CE-CS-L were slightly higher than that of CS-M and CE-CS-M. Compared to the composite films incorporated with CS-M, it was shown that the composite films incorporated with CS-L have lower tensile elongation but higher tensile strength and higher modulus, as well as higher heat resistance and lower weight loss. Previously, it was shown that the addition of chitosan in starch-based biodegradable composite films decreased elongation at break but improved tensile strength and tensile modulus as well as increased the thermal stability of the composite films [41]. The CE/CS/NR composite films presented increased thermal stability when compared with CE and CS. With good distribution and dispersion of CE and CS in the NR matrix, the interfacial adhesion between the fibers and the NR matrix could be enhanced, resulting in more interaction between CE, CS, and NR and improved thermal stability. Similar TGA patterns of NR, CE, and their composites have previously been reported on the composite films of NR reinforced with cellulose using a latex aqueous microdispersion process [13,28,29].

The DTG curves show the percent derivative weight of CE, CS, NR, and CE/CS/NR composite films at different heating rates in nitrogen atmosphere (Figure 5b). Under thermal analysis, all samples showed main degradation stages at 200–500 °C. The decomposition of the NR and CE/CS/NR composite films occurs in a single step, and the decomposition of CE, CS-L, and CS-M occurs in two steps. At the initial step, the percent derivative weights of CE, CS-L, and CS-M are around 2%/min at a temperature of approximately 100 °C, which is associated with the vaporization of volatile components, such as moisture. In the second step, the main decomposition of CS-L and CS-M occurs at around 297.4 °C and 293.3 °C, respectively, which is assigned to depolymerization reaction. On the other hand, the DTG maxima temperature in the case of CE and NR occurs at 351.4 °C and 379.5 °C, which is higher than that of CS. This indicates that the amino polymer is less thermally stable than the hydrocarbon polymer. The parameter maximum weight loss rate (MWLR) for NR is highest (18% min^−1^), followed by CE (14% min^−1^) and CS (8–9% min^−1^), respectively. The values of MWLR for CE/CS/NR composites lies between those of NR, CE, and CS.

### 3.5. X-ray Diffraction (XRD) 

**Figure 6:**
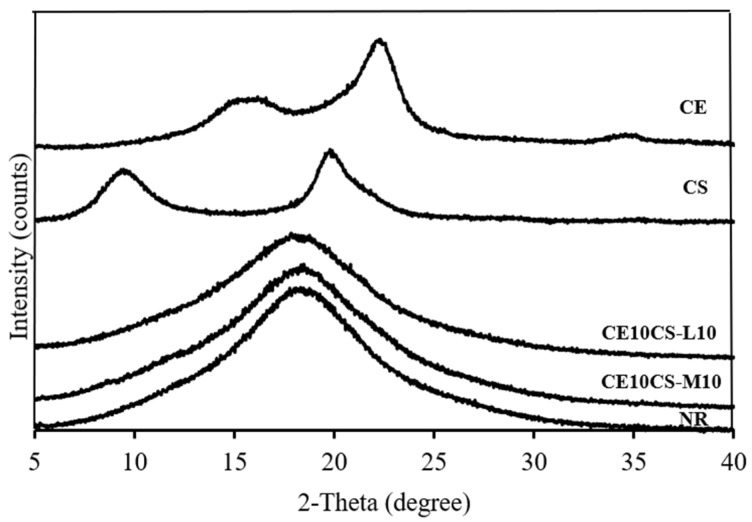
XRD patterns of NR, CE, CS, CE10CS-L10, and CE10CS-M10 films.

The material crystallinity was determined by XRD analysis. The XRD patterns of NR, CE, CS-L, CS-M, and CE/CS/NR composite films are shown in Figure 6. The characteristic peaks of CE comprise two main peaks at two theta angles (2θ) around 16.3° and 22.3°, corresponding to 110 and 200 reflection planes, respectively, which are the typical patterns of cellulose I allomorphs [29,42,43]. The XRD pattern of CS shows characteristic peaks at two theta angles around 9.9° and 19.8°, corresponding to the (020) crystalline planes and (200) reflection planes, respectively [44,45]. The NR film displays a typical diffraction pattern of an amorphous polymer with a significant broad peak located at a two-theta angle of 18.3° [46,47]. Corresponding to the structure containing NR as a major component, the XRD patterns of the composite films of CE10CS-L10 and CE10CS-M10 showed a typical diffraction pattern of an amorphous polymer with a significant broad peak at a two-theta angle around 18.3° and 18.1°, respectively, which is a similar diffraction pattern compared to the NR film. However, no diffraction peaks of CE and CS were observed. The disappearance of CE and CS peaks might be due to the low concentrations of CE and CS in the composite films and interactions among the components of CE, CS, and NR in the composite films [29,48]. 

NR, CE, CS-L, and CS-M have crystallinity values at 0%, 62%, 76%, and 63%, respectively. The degree of crystallinity of CE10CS-M10 is 0.7%, whereas the crystallinity of CE10CS-L10 is ~0%. The loss of crystalline nature of CE and CS in the composite films could be due to hydrogen bonding between NR, CE, and CS, which allows parallel chain arrangement in the crystals of CE and CS, which impacts the crystalline arrangement of CF and CS in the composite films [29,48]. As a result, the composite films of CE10CS-L10 and CE10CS-M10 have a very low degree of crystallinity, similar to that of the NR film. 

### 3.6. Contact Angle

The material hydrophilicity was determined by measuring the contact angle of the films, as shown in Table 2. Generally, on a hydrophilic surface, the contact angle will be less than 90°. The contact angles of pure NR, CE, CS-M, and CS-L films are 102.2°, 74.5°, 94.8°, and 91.2°, respectively. NR is a hydrophobic substance due to its long hydrocarbon polymer chains. The dynamic contact angle of the NR composites decreases with the addition of CE and CS. The increase in the number of surface hydroxyl groups from the addition of CE and CS enhances the hydrophilicity of the films. In addition, the contact angle might also decrease because of the increase in external surface roughness [49].

The contact angles of the composite films of CE10CS-L10 and CE10CS-M10 decreased to 88.3° and 82.3°, respectively. Cellulose and chitosan have vast amounts of hydroxyl groups. Therefore, NR composite films containing CE and CS show significantly enhanced hydrophilicity. Consequently, reinforcement with CE and CS would affect water absorption capacity and solubility in polar and nonpolar solvents.

### 3.7. Water Absorption Capacity (WAC)

**Figure 7:**
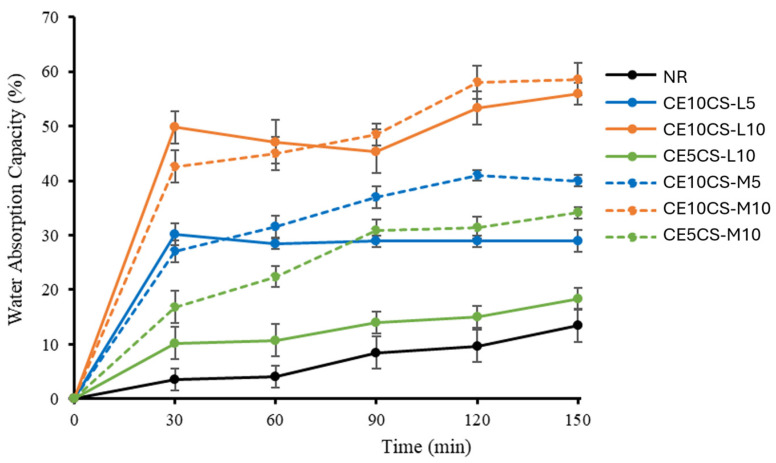
Water absorption capacity (WAC%) with time of NR and CE/CS/NR composite films.

The measurement of water absorption capacities (WAC) of NR and CE/CS/NR films was carried out with respect to the immersion time in the range of 0–150 min (Figure 7). The reinforcement of CE and CS at 5–10% wt. in the NR matrix enhanced the rate of water absorption. During the 30 min of immersion, water was rapidly absorbed into CE10CS-L10, CE10CS-M10, CE10CS-L5, CE10CS-M5, CE5CS-L10, and CE5CS-M10 films, reaching equilibrium condition at around 150 min. The pure NR film slowly absorbed water, and the WAC at 150 min was only 13.4% due to the hydrophobic nature of NR, whereas the films CE10CS-L10 and CE10CS-M10 had considerably higher WAC at around 56 and 58%, respectively. The CE10CS-M10 had more WAC than CE10CS-L10 because CS-M could form a relatively higher number of hydroxyl groups as compared to CS-L [34]. In a similar way, the film of CE5CS-M10 has higher WAC (27%) compared to CE5CS-L10 (14%).

The WAC of the composite films increased with an increase in CE and CS content because CE and CS are hydrophilic and able to form hydrogen bonds with water. According to the chemical structure of CE and CS, the increase in CE and CS content in the composites could increase the amount of hydrogen bonding networks between water molecules and both hydroxyl and amine groups [50,51]. According to the porous structure of CE, water molecules could be physically trapped in the delicate network structure of CE. CS is also a porous material. In this study, by using CS in solution form for the dispersion of CE powders in NRL, the WAC of the composite films increased with the increase in CS content from 5% to 10%. However, it was demonstrated that CE has a greater influence on WAC than CS, because the integration of CE powders in the NR matrix might create greater voids, resulting in more water absorbed into the composite film. Nevertheless, the incorporation of CS in other systems might make the film structure more compact [39,50].

### 3.8. Toluene Uptake

**Figure 8:**
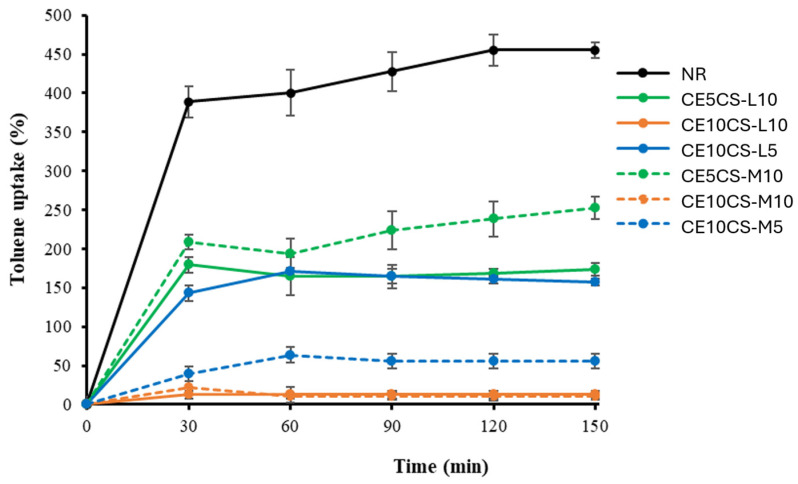
Toluene uptake with time of NR and CE/CS/NR composite films.

Toluene is widely used in industry as a nonpolar solvent, such as its usage as a solvent for paints, coatings, adhesives, and rubber products. In this study, toluene was used as a non-polar solvent in order to measure the film’s ability to absorb a non-polar solvent (or toluene uptake, TU). The stability of the films in toluene was observed as well. The TU of NR and CE/CS/NR films was carried out with respect to the immersion time of 0–150 min (Figure 8). The TU of the NR film rapidly increased and reached the maximum value at 450% within 120 min. After that, the NR film dissolved almost completely in toluene within 4 h. NR and toluene are nonpolar. Thus, this solvent is highly absorbed into NR film, and the NR film could dissolve in toluene. The reinforcement of CE and CS in the NR matrix enhanced the hydrophilic property of the films; therefore, the films of CE10CS-L10 and CE10CS-M10 showed considerably improved resistance to non-polar solvents such as toluene and exhibited very low TU at around 12.5 and 13.33%, respectively. No significant structural change of the CE10CS-L10 and CE10CS-ML10 films were observed during the immersion in toluene for 4 h. Overall, the TU of CE/CS/NR composite films decreased with the addition of CE and CS into the NR matrix. Increasing CE and CS content in NR composite films resulted in increasing hydroxyl and amine groups, which enhanced the hydrophilic property of the composite films, resulting in significantly improved non-polar solvent resistance [50].

### 3.9. Antimicrobial Ability

**Figure 9:**
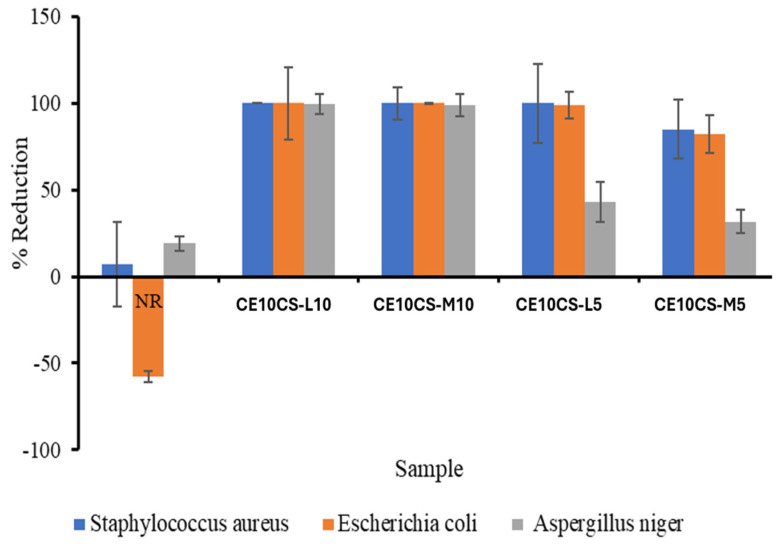
Reduction of *S. aureus* (■), *E. coli* (■), and *A. niger* (■) from the antimicrobial efficacy validation of NR and CE/CS/NR composite films.

*E. coli* (gram negative bacteria) and *S. aureus* (gram positive bacteria) were used for the antibacterial tests, and *A. niger* was used for the antifungal test. The reinforcement of CE and CS at 5–10% wt. in the NR matrix significantly enhanced antimicrobial ability of the composite films. Generally, CS can effectively control the growth and reproduction of hazardous bacteria and exhibits antibacterial activities on both Gram-positive and Gram-negative bacteria. The antibacterial activity of CS was found to be greater on *S. aureus* than on *E. coli* [52]. The results, as shown in Figure 9, revealed the antimicrobial effect of the integration of CS of MW 30,000 (CS-L) and 500,000 (CS-M) into the composite films. The maximum antimicrobial activity against *S. aureus*, *E. coli*, and *A. niger* at about 100% was obtained from the composite films of CE10CS-L10 and CE10CS-M10. In this study, it was demonstrated that, to some extent, CS-L could inhibit microorganisms more effectively than CS-M. In addition, the percentage of reduction increased with increasing CS-L and CS-M content from 5 to 10% wt. The antimicrobial ability of composite films composed with CS should depend on their cationic nature and molecular size. Previously, from the study of the effect of the antimicrobial activities of CS against *E. coli*, it was reported that CS samples with MWs from 5.5 × 10^4^ to 15.5 × 10^4^ Da had antimicrobial activities at concentrations higher than 200 ppm, and the antibacterial activity of low MW chitosan was higher than that of the high MW chitosan [53]. The presence of CS in the biocomposite of CS and NR also resulted in enhanced antibacterial activity against *S. aureus* [38]. CS also has a strong antifungal effect, and it has been used to directly inhibit the growth of several fungal pathogens on crop plants [54]. A slight reduction effect was observed in the system incubated with *S. aureus* and *A. niger* on NR film, while the number of *E. coli* increased. The negative percentage reduction for NR indicated an increased number of *E. coli* in the system incubated with the NR film. Hence, the NR sample did not have antibacterial effects against *E. coli*.

## 4. Conclusions

The CE/CS/NR composite films were successfully prepared via latex aqueous microdispersion. The reinforcement of CE and CS at 5–10% wt. dispersed homogeneously within the NR matrix. The mechanical properties were effectively enhanced by the reinforcement of CE and CS. The highest tensile strength and Young’s modulus, approximately 13.8 MPa and 12.7 MPa, respectively, were obtained from the composite film of CE10CS-L10, while the composite film of CE10CS-M5 demonstrated high elastic elongation at 526%. The composite film demonstrated an improvement in thermal properties. The hydrophilicity of the composite films increased when CE and CS were added to the NR matrix. In addition, due to the interfacial interactions between CE, CS, and NR, the structural and chemical stability were improved. The presence of CS in the CE/CS/NR composites resulted in enhanced antibacterial activities against *S. aureus* and *E. coli* and antifungal activity against *A. niger*. The maximum antimicrobial inhibition, with the reduction of *S. aureus*, *E. coli,* and *A. niger* by 100%, was obtained from CE10CS-L10 and CE10CS-M10. To the best of our knowledge, this type of combination (CE/CS/NR) is reported for the first time in this work, and the results demonstrate that the CE/CS/NR composite films are promising NR-based products of green, environmentally friendly materials for elastic packaging.

## Figures and Tables

**Table 1:** Compounding formulations for NR/CE/CS composites.

Samples	CE (g)	CS (g)		NRL (60% DRC) (g)
		CS-L	CS-M	
NR	-	-	-	6
CE5CS-L5	0.18	0.18	-	6
CE5CS-M5	0.18	-	0.18	6
CE5CS-L10	0.18	0.36	-	6
CE5CS-M10	0.18	-	0.36	6
CE10CS-L5	0.36	0.18	-	6
CE10CS-M5	0.36	-	0.18	6
CE10CS-L10	0.36	0.36	-	6
CE10CS-M10	0.36	-	0.36	6

**Table 2:** Degree of dynamic water contact angles (°) of NR, CE, CS-M, CS-L, and NR composites.

NR	CE	CS-M	CS-L	CE10 CS-L5	CE10 CS-M5	CE10 CS-L10	CE10 CS-M10	CE5 CS-L10	CE5 CS-M10
102.2 ± 1.2	74.5 ± 1.8	94.8 ± 2.8	91.2 ± 4.7	96.8 ± 4.7	95.7 ± 4.7	88.3 ± 6.4	82.3 ± 3.0	94.2 ± 2.6	95.2 ± 5.1
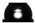	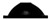	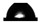	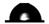	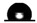	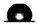	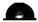	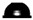	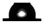	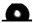

## Data Availability

The original contributions presented in the study are included in the article/Supplementary Materials, further inquiries can be directed to the corresponding author.

## Supplementary Materials

The following supporting information can be downloaded at: https://www.mdpi.com/article/10.3390/polym16182652/s1, Table S1: The technical sheet for chitosan (CS-L and CS-M).
