# Supplementary material for: Natural Rubber Films Reinforced with Cellulose and Chitosan Prepared by Latex Aqueous Microdispersion
Source: Polymers (Basel). 2024 Sep 20;16(18):2652. doi: 10.3390/polym16182652 (PMC11435537; doi:10.3390/polym16182652)
Supplement: Supplementary file 1 [file polymers-16-02652-s001.zip › polymers-3168041-supplementary.pdf]

# The technical sheet for chitosan (CS-L and CS-M)

## TS Agritech

**ที่อยู่ :** เลขที่ 100/89 **ม.4 เกษมเพชรวิลล่า เขต : ลำพยา แขวง : เมือง**
**จังหวัด : นครปฐม** **รหัสไปรษณีย์** :73000
**เบอร์โทร :** 034-986656 **มือถือ :** 081-0992402 **และ** 086-7986109
**อีเมล :** saha6346@gmail.com
**เว็บไซต์ :** www.tschitosan.com

---

## Chitosan

| Parameters | Description |
|---|---|
| Appearance (powder) | White or yellow |
| Particle size | 1-1.5 mm |
| Moisture content (%) | 13.02 ± 0.01 |
| Solubility (%) | 98.42 ± 0.04 |
| Insoluble matters (%) | 1.58 ± 0.04 |
| Protein content (%) | 1.27 ± 0.07 |
| Degree of deacetylation (%) | 90.21 ± 0.05 |
| Ash content (%) | 1.78 ± 0.06 |
| pH | 6.5-7.5 |
| Mw (CS-L) | 30,000 - 50,000 |
| Mw (CS-M) | 300,000- 500,000 |
